# Improvement of Mechanical Property for PLA/TPU Blend by Adding PLA-TPU Copolymers Prepared via In Situ Ring-Opening Polymerization

**DOI:** 10.3390/polym14081530

**Published:** 2022-04-09

**Authors:** Hui Fang, Lingjie Zhang, Anlin Chen, Fangjuan Wu

**Affiliations:** 1 College of Materials Science and Engineering, Fujian University of Technology, Fuzhou 350118, China; hfang79@163.com (H.F.); ljzhangfhcl@163.com (L.Z.); alchenfhcl@163.com (A.C.); 2 Key Laboratory of Polymer Materials and Products of Universities in Fujian, Fujian University of Technology, Fuzhou 350011, China; 3 Fujian Provincial Key Laboratory of Advanced Materials Processing and Application, Fujian University of Technology, Fuzhou 350011, China

**Keywords:** polylactic acid, thermoplastic polyurethane, in situ polymerization, compatibility

## Abstract

Polylactic acid (PLA)-thermoplastic polyurethane (TPU) copolymer (PTC) was prepared by melting TPU pellets in molten lactide, followed by in situ ring-opening coordination polymerization. The results from FTIR and ^1^H-NMR confirmed the formation of the copolymer. PLA/TPU blends with different TPU contents were prepared by melt blending method. SEM and mechanical properties showed a conspicuous phase separation between PLA and TPU. In order to further improve the mechanical properties of the blend, PTC was used as the compatibilizer and the effects of the PTC content on the properties of the blend were investigated. The addition of PTC made TPU particles smaller in PLA matrix and improved the compatibility. With the loading of 5 wt.% PTC, the impact strength of the PLA/TPU blend reached 27.8 kJ/m^2^, which was 31.1% and 68.5% higher than that of the blend without PTC and pure PLA, respectively. As the content of PTC was more than 5 wt.%, the mechanical properties declined since the compatibilizer tended to form separate clusters, which could reduce the part distributed between the dispersed phase and the matrix, leading to a reduction in the compatibility of the blend. Moreover, the DMA results confirmed PTC could improve the compatibility between PLA and TPU.

## 1. Introduction

Owing to the low production cost, excellent physical properties, and good thermal stability, polymers produced by the petroleum industry are favored. However, the environmental pollution and rapid consumption of fossil fuels caused by the extensive use of petroleum-based polymers and improper treatment have become a global environmental problem [1,2]. With the increasing awareness of environmental protection and the trend of reducing the use of fossil fuels, biodegradable polymers, especially those derived from renewable resources, have attracted increasing attention in recent years [3]. The bio-based polymer material presents a new material with excellent performance and no pollution to the environment. It originates from natural resources and can be gradually degraded in the natural environment, and finally returns to nature in the form of water, carbon dioxide, and other small molecules, which can ultimately reduce the consumption of petroleum-based polymers and carbon dioxide emissions [4]. Biodegradable polymers include polylactic acid (PLA), polycaprolactone (PCL), polybutylenes (PBS), poly(hydroxy alkanoates) (PHAs), poly(3-hydroxybutyrate) (PHB), and polyesteramide (PEA), etc. [5,6,7]. Among these polymers, PLA has been widely used in biomedicine, antibacterial materials, packaging, and auto parts [8,9,10,11,12] due to the advantages of high strength, high modulus, good biocompatibility, and easy processing [13,14].

PLA can be obtained by direct condensation of lactic acid and ring-opening polymerization of cyclic dimer lactide through fermentation of sugars extracted from renewable resources such as corn, sugar cane, wheat, sugar beet, and potato [15]. Since direct polycondensation is a reversible reaction involving chemical equilibrium, it is hard to remove trace water in the polymerization process, and thus the reaction cannot proceed forward. Therefore, the ring-opening polymerization of lactide in the presence of a catalyst represents the major source of PLA with a high molecular weight obtained [16]. PLA has good physical properties and a wide range of processing temperatures, which can be used in extrusion spinning, thermal forming, and other processing methods [2]. Although PLA has shown many advantages and a wide range of applications, it still has some defects, and its inherent brittleness, low impact resistance, and flexibility limit its further application [17]. PLA could be strengthened by many polymers, such as poly(ethylene succinate) (PES), poly(butylene succinate) (PBS), poly(ethylene glycol) (PEG), PCL, Poly(3-hydroxybutyrate) (PHB), poly(Butylene adipate-co-terephthalate) (PBAT), and thermoplastic polyurethane (TPU) [18,19,20,21]. Among these polymers, TPU has attracted much attention due to its excellent flexibility, good biocompatibility, bio-stability, wear-resistant durability, and smoothness [22,23,24].

Various methods, such as melting blending [25], solvent casting [26], electrostatic spinning [27], and fused deposition modeling [28], have been used to prepare PLA/TPU blends to improve the flexibility of PLA and further expand its application range. As a traditional and convenient processing method, melt blending has attracted more attention [29]. Xin et al. [30] prepared biodegradable PLA/TPU blends by twin-screw extrusion. With the addition of TPU, PLA changes from brittle fracture to ductile fracture. When the TPU content measures 40 wt.%, the elongation at break increases to 400%. The research of Sharifah et al. [31] showed that the elongation at break of PLA increased significantly with the incorporation of 50 wt.% TPU but both modulus and strength decreased significantly. Han et al. [32] reported that when the TPU content exceeded 30 wt.%, the tensile strength gradually decreased with the increase of TPU content. Although there is compatibility between PLA and TPU elastomer soft segments, PLA and TPU are immiscible. To significantly toughen PLA, it is necessary to add a large amount of TPU, which inevitably leads to a significant decrease in tensile strength and Young’s modulus. In order to overcome this shortcoming, some compatibilizers have been used to improve the compatibilization of PLA and TPU. Compatibilizers commonly used to improve PLA/TPU blends include hydrophobic silica nanoparticles, 1,4-phenylene diisocyanate, methylenediphenyl diisocyanate, ethylene-methyl acrylate glycidyl methacrylate copolymer, etc. [33,34,35,36] Moreover, compatibilizers identical with a component of PLA/TPU blend were used in some studies to enhance compatibilization, thus maintaining the biocompatibility and biodegradability of PLA and TPU. For example, Zhang et al. [37] used a small amount of PUEP as a reactivity compatibilizer for PLA and TPU components, and the experimental results showed that the mechanical properties of PLA/TPU/PUEP blends were significantly improved. In particular, the notch impact strength increased by nearly 8 times, with its toughening effect owing to the reaction between the reactive group -NCO in PUEP and the -OH of PLA and PU at the same time, thus enhancing the interface interaction. Mo et al. [38] prepared a PLA-g-TPU graft copolymer by the melting blending method as a compatibilizer of the PLA/TPU blend, and the experimental results showed that the addition of the compatibilizer significantly improved the mechanical properties of PLA/TPU blend. Thus far, many compatibilizers have been used for PLA/TPU blends, and good progress has been made. However, most of them are toxic, thus it is hard to find usage in the medical field.

In this work, L-lactide was first used as raw material to dissolve TPU under heating conditions, and PLA-TPU copolymer was prepared by in situ ring-opening polymerization, named PTC. The impact strength of PLA/TPU blend was significantly improved when the synthesized PTC was used as the compatibilizer of PLA/TPU blends, compared with the PLA/TPU blend without PTC. This work is expected to provide a novel strategy for the preparation of biological PLA/TPU blends with good properties.

## 2. Experimental

### 2.1. Materials

L-lactide (LA) was purchased from Corbion with a purity of L-isomer > 99.5%. PLA (3040D) was manufactured by Nature-Works (Minneapolis, MN, USA). TPU (DESMOPAN DP 9370A) was purchased from Bayer Co., Ltd., Leverkusen, Germany. Tin 2-ethylhexanoate (Sn(Oct)_2_) (95% purity), 1, 4-butanediol (BDO) (95% purity), and tris(2,4-di-ter-butylphenyl) phosphite (AO168) (98% purity) were purchased from Aladdin Reagents Ltd., Pico Rivera, CA, USA. Both chloroform and methanol with the purity of 99% were purchased from Sinopharm Chemical Reagents Co., Ltd., Shanghai, China.

### 2.2. Preparation of PLA-TPU Copolymer

**Figure 1:**
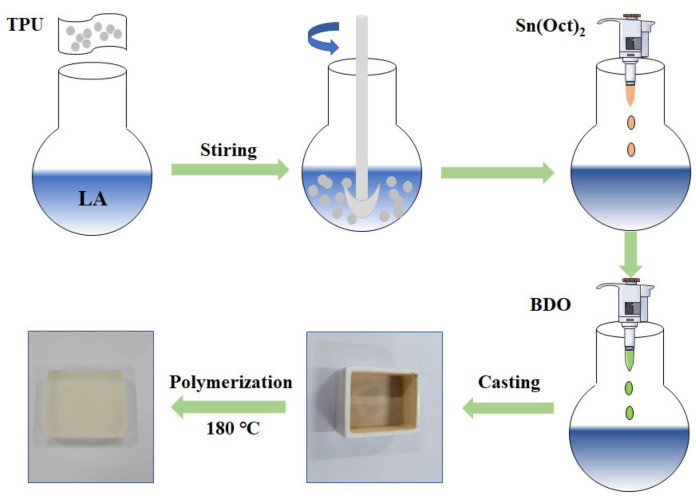
Preparation process of PLA-TPU copolymer.

PLA-TPU copolymer (PTC) was prepared by in situ ring-opening coordination polymerization. Firstly, quantified TPU (10 wt.% of LA) was dissolved in LA with mechanical stirring at 120 °C. After 2 h, a homogenous solution was obtained. Then, a certain amount of AO168 and Sn(Oct)_2_ were added and the mixture was vacuumed for 15 min to remove trace moisture. BDO was successively added in the mixture under nitrogen. The fully mixed mixture was poured into preheated mold and polymerized at 180 °C for one hour to obtain PTC. The preparation process is shown in Figure 1.

### 2.3. Preparation of PLA/TPU Blends

PLA/TPU blends were prepared by melt-blending method. PLA, TPU and PTC were vacuum-dried for 8 h, and then the samples were mixed in different proportions in a HAAKE mixer with a constant speed of 60 rpm at 180 °C for 10 min. In order to investigate the influence of PTC on PLA/TPU blends and eliminate the effect of different TPU content on the properties, the proportion of PLA and TPU was fixed as 90:10. PLA/TPU blends were prepared by the same method taking PTC as the compatibilizer. The obtained samples were recorded as PT10/PTC2.5, PT10/PTC5, PT10/PTC7.5, PT10/PTC10. The formulation of PLA/TPU blends and PLA/TPU/PTC blends are summarized in Table 1.

### 2.4. Purification of PTC

To investigate the copolymerization of TPU and PLA, the PTC was purified with chloroform. Briefly, a piece of PTC was dissolved in chloroform upon stirring. After several hours, the sample was completely dissolved, which indicated that TPU was non-existent in the sample due to the insolubility of TPU in chloroform. The obtained homogeneous solution was poured into anhydrous methanol, and then white solid precipitated. The white solid was filtered and washed with methanol several times. This procedure was repeated three times to exclude unreacted monomers and oligomers. Finally, the white solid was dried in a vacuum oven at 80 °C for 8 h and was noted as purified-PCT.

### 2.5. Characterization

Fourier transform-infrared (FTIR) spectroscopy (Nicolet 6700, Thermo Fisher, Waltham, MA, USA) was used to collect the infrared spectral data of the samples in the range of 500–4000 cm^−1^, and the structure of the samples was analyzed and characterized. The PTC copolymer was dissolved in deuterium chloroform with tetramethylsilane as internal standard. ^1^H-NMR spectra were recorded on a Bruker Advance NEO 600 MHz (14.1 T) spectrometer. Field emission scanning electron microscope (FESEM, NovanoSEM450, FEI Company, Hillsboro, OR, USA) observed the microscopic morphology of the samples, and the acceleration voltage was tested at 5 kV. All samples were fractured by liquid nitrogen to obtain smooth and flat surfaces, and the cross sections were treated with gold spraying at 5 nm. The dynamic thermo-mechanical properties of the samples were studied using a dynamic thermo-mechanical analyzer (DMA242E, Netzsch, Selb, Germany). Using single cantilever mode, rectangular splines were injected by Hacker microinjection molding machine with a width and thickness of 10 mm and 4 mm, respectively, and cantilever spacing of 16 mm. The test conditions were: heating rate in the heating chamber was 3 °C/min, test temperature range was −70 °C to 120 °C, final test frequency was constant 1 Hz, and the dynamic force was 4 N. The tensile properties of the samples were tested by universal tensile testing machine (AGS-X, Shenzhen, China), in which the stretching dumbbell splines were prepared by HAAKE Microinjection molding machine (Thermo Fisher, Waltham, MA, USA) according to ISO527-2-5A standard. The sample was stretched at a rate of 10 mm/min. The impact strength of non-notched samples was tested by pendulum impact tester (JB-300B, Haida, Jinan, China). The pendulum adopts the simply supported beam mode, and the impact energy was 7.5 J.

## 3. Results and Discussion

### 3.1. FTIR Analysis

**Figure 2:**
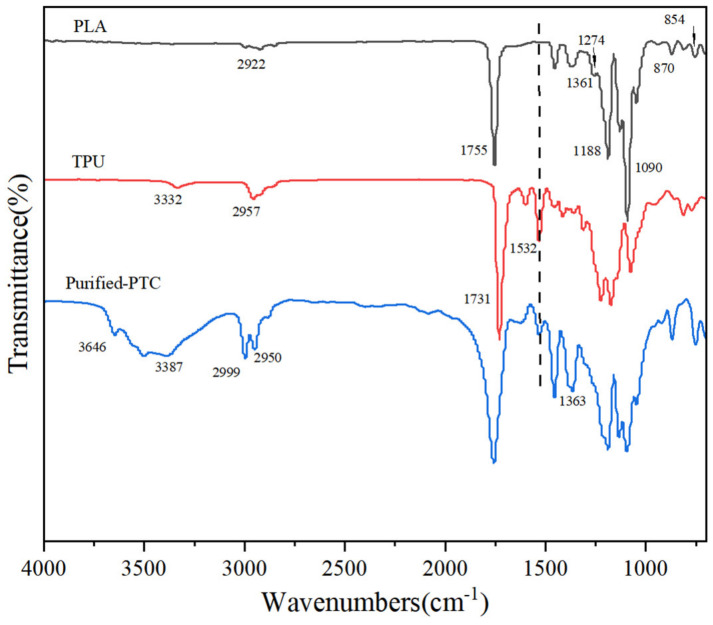
FTIR spectrum of PLA, TPU and purified-PTC.

The chemical structure of PLA, TPU, and purified-PTC was analyzed by FTIR spectroscopy and the results are shown in Figure 2.

The peaks in PLA at 2922 cm^−1^, 1754 cm^−1^, 1360 cm^−1^, 1274 cm^−1^ and 1090 cm^−1^ were ascribed to the tensile vibration of -CH_3_, the tensile vibration of -C = O, the bending vibration of -C-H, the bending vibration of -CH_3_, and tensile vibration of C-C(O)-O, respectively [39]. The peaks in TPU at 3646 cm^−1^, 3332 cm^−1^, 2956 cm^−1^, 1730 cm^−1^, and 1532 cm^−1^ corresponded to -O-H, -N-H, -C-H, -C = O tensile vibration, and -N-H bending vibration, respectively [38]. Furthermore, the bending vibration of -N-H at 1532 cm^−1^ was discovered in the spectrum of purified-PCT. Meanwhile, the characterization peaks of PLA were also discovered. This indicated that TPU might have reacted with PLA during the ring-opening polymerization.

### 3.2. H-NMR Analysis

**Figure 3:**
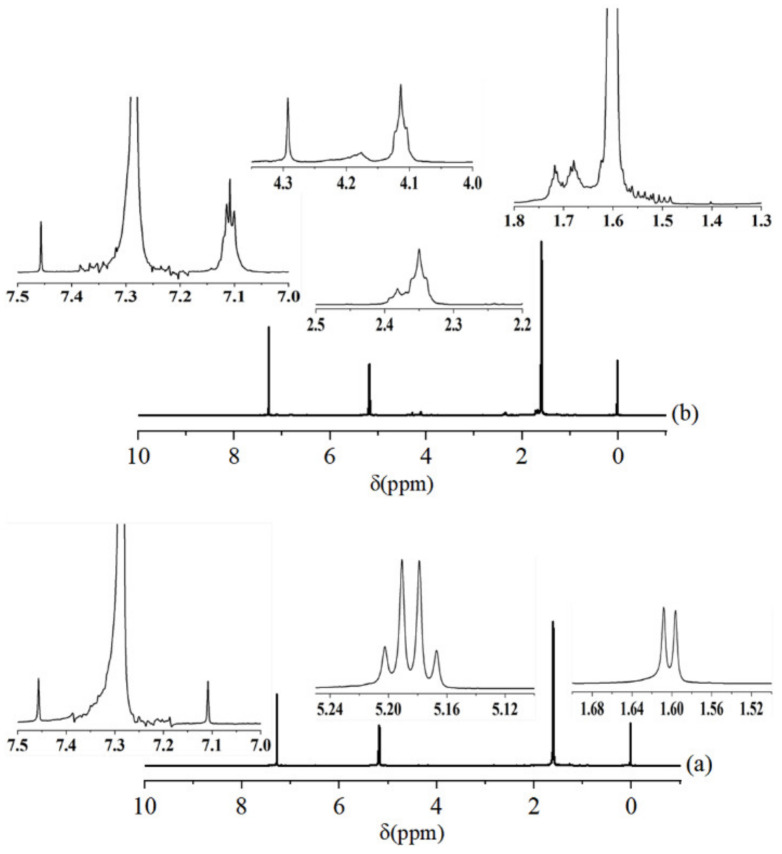
^1^H-NMR spectra of (**a**) PLA and (**b**) purified-PTC.

^1^H-NMR was used to further study the structure of PLA and purified-PCT, and the results are shown in Figure 3.

**Figure 4:**
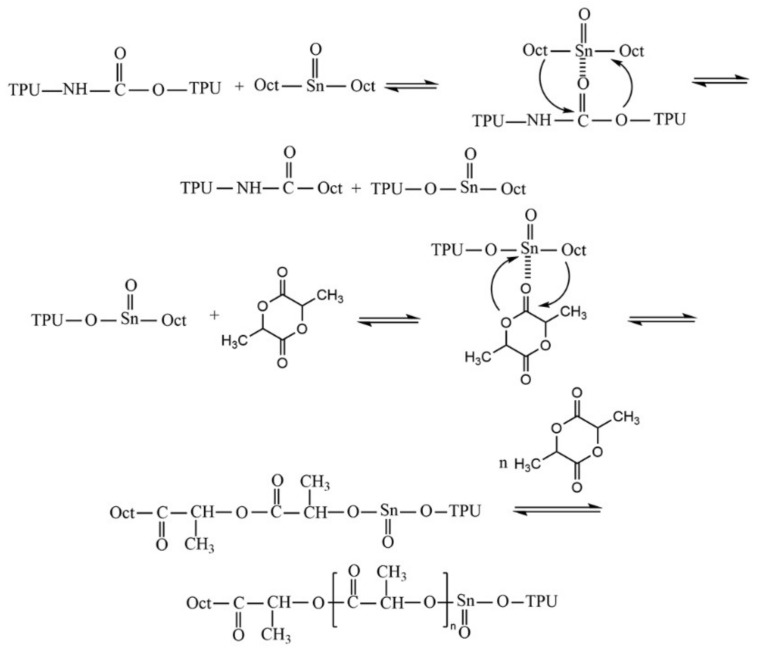
Probable reaction mechanism of PLA/TPU copolymer.

As can be seen from Figure 3a, the characteristic peaks at 1.59 ppm (3H), were assigned to the chemical shift of the H of the methyl-methyne (CH_3_-CH) groups. The perfect quartet peaks at 5.20 ppm (1H) were assigned to the chemical shift of the H of the methyne (-CH-CH_3_) groups. ^1^H-NMR spectra of PLA were in complete accordance with the anticipated chemical structure for PLA [40]. Moreover, two very weak characteristic peaks representing the chemical shift of PLA hydroxyl proton were observed at 7.10 ppm and 7.45 ppm. Meanwhile, the peak at 7.29 ppm was assigned to the chemical shift of the H impurities of CDCl_3_. Figure 3b shows the ^1^H-NMR spectra of purified-PCT, which were extracted in CDCl_3_ from PTC. Compared with the PLA spectra, the peaks of purified-PCT in the 1.66–1.72 ppm region, at 2.36 ppm and in the 3.89–4.38 ppm region corresponded to -CH_2_ in TPU main chains and -O-CH_2_ adjacent to the urethane group, respectively [41]. Moreover, the single peak at 7.10 ppm changed to multiple peaks, which indicated that the TPU had reacted with PLA hydroxyl proton. Hence, the above results confirmed the formation of PLA-g-TPU copolymers during the in situ ring opening polymerization. The probable reaction mechanism is shown in Figure 4.

### 3.3. Morphology

**Figure 5:**
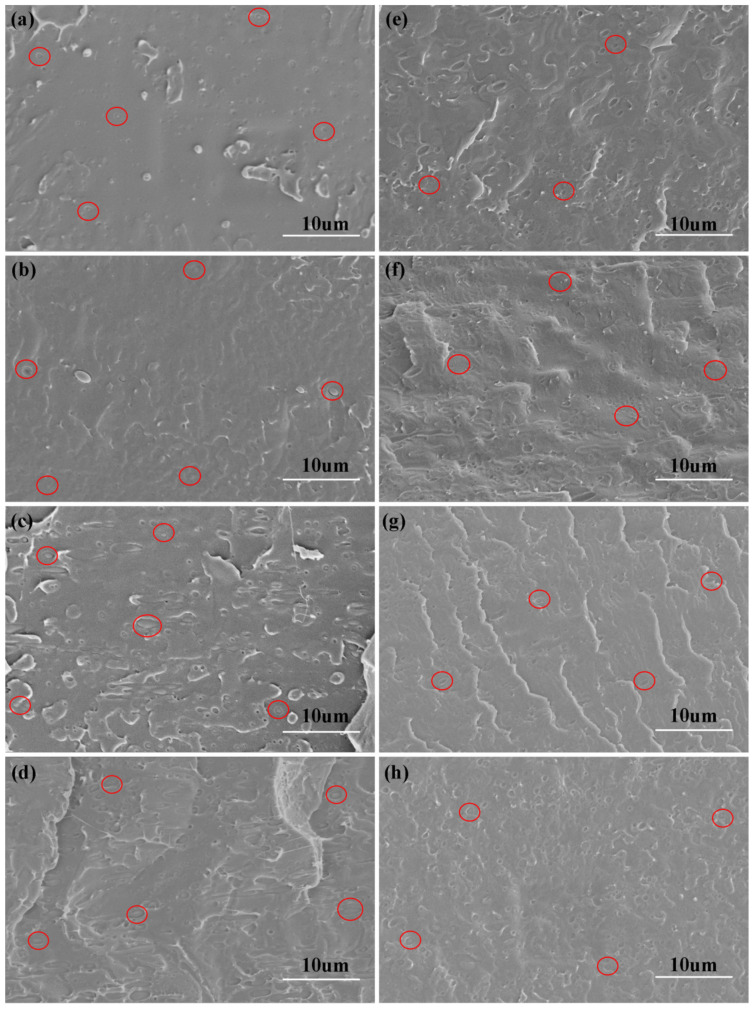
FESEM graphs of the fractured surface of (**a**) PT1, (**b**) PT3, (**c**) PT5, (**d**) PT10, (**e**) PT10/PTC2.5, (**f**) PT10/PTC5, (**g**) PT10/PTC7.5, and (**h**) PT10/PTC10.

The morphology of PLA/TPU and PLA/TPU/PTC blends was studied by FESEM, and the dispersion of TPU in PLA matrix was observed. The results are shown in Figure 5. As shown in Figure 5a–d, the TPU microspheres randomly dispersed in the PLA continuous phase. As the TPU content increases, the diameters of TPU microspheres increase. Moreover, irregular shapes of TPU dispersed phases (especially in the SEM graph of PT10) could be seen, which was possibly due to the increase in viscosity of the system. Moreover, the surface of TPU phase was smooth due to weak interface adhesion between the domains and continuous phase.

On the contrary, as shown in the SEM graphs of PLA/TPU/PTC blends, a more homogeneous morphology could be observed due to the incorporation of PTC as a compatibilizer. The average diameter of the TPU microspheres decreased sharply and particle size distribution became more homogenous. The diameter reduction of TPU was attributed to the reduced surface tension and improved interfacial adhesion between PLA and TPU phases [42]. When the content of PTC reached 5.0 wt. %, it can be seen from Figure 5f that there was no obvious interface between TPU particles and PLA matrix, indicating that PTC as a compatibilizer improved the interfacial compatibility of the PLA/TPU blend. When the PTC content further increased to 7.5 wt.% and 10.0 wt.%, although the compatibility of the blends was improved, the high content of filler was unevenly dispersed in the polymer matrix, resulting in the decline of mechanical properties, which is consistent with the mechanical properties and DMA results.

### 3.4. Mechanical Property

Table 2 lists the mechanical properties of PLA/TPU blends and PLA/TPU/PTC blends.

As shown, the tensile strength and Young’s modulus of PLA/TPU blends decreased with an increase of TPU content. When the TPU content was 10 wt.%, the tensile strength and the Young’s modulus decreased from 65.2 MPa and 5567 MPa to 58.6 MPa and 4748 MPa, respectively, but the impact strength increased from 16.5 kJ/m^2^ to 21.2 kJ/m^2^. It suggested that incorporating TPU could promote the toughness of PLA but reduced the strength and stiffness, which is consistent with the results in the reported works.

To promote the mechanical properties of the PLA/TPU blend, the PTC as the compatibilizer was applied. It can be seen from Table 2 that the addition of PTC made the tensile strength and Young’s modulus of all PLA/TPU/PTC blends higher than those of PT10. The impact strength increased first and then decreased with the increase of PTC content. When the PTC content was 5 wt.%, the impact strength of PT10/PTC5 blend reached the maximum value of 27.8 kJ/m^2^, which is 6.6 kJ/m^2^ higher than that of PT10, increasing by 31.1%. Consequently, adding PTC could significantly enhance the toughness of the blend. There are some similar results from other research [43,44]. In Mahmud et al. ‘s study [43], when the addition amount of TPU was 25 wt.%, the impact strength of PLA/TPU blend increased from 5.46 kJ/mm^2^ of pure PLA to 6.54 kJ/mm^2^, with a very limited increase in impact strength. When the amount of TPU increases to 50 wt.%, the impact strength of PLA/TPU blends increases to 55.81 kJ/mm^2^. When 5 phr EMA-GMA was added, the impact strength increased from 55.8 kJ/mm^2^ to 165.8 kJ/mm^2^.

However, when the content of PTC reached 10 wt.%, the impact strength of PT10/PTC10 decreased to 21.0 kJ/m^2^. As the content of compatibilizer was excessive, the compatibilizer tended to form separate clusters, which could reduce the part distributed between the dispersed phase and the matrix, leading to the reduction in compatibility of the blend. Hence, the impact strength of the PLA/TPU/PTC blends declined when the content of PTC was more than 5 wt.%. Generally, the addition of PTC improved the toughness of the blends without reducing their strength and stiffness.

### 3.5. DMA Analysis

**Figure 6:**
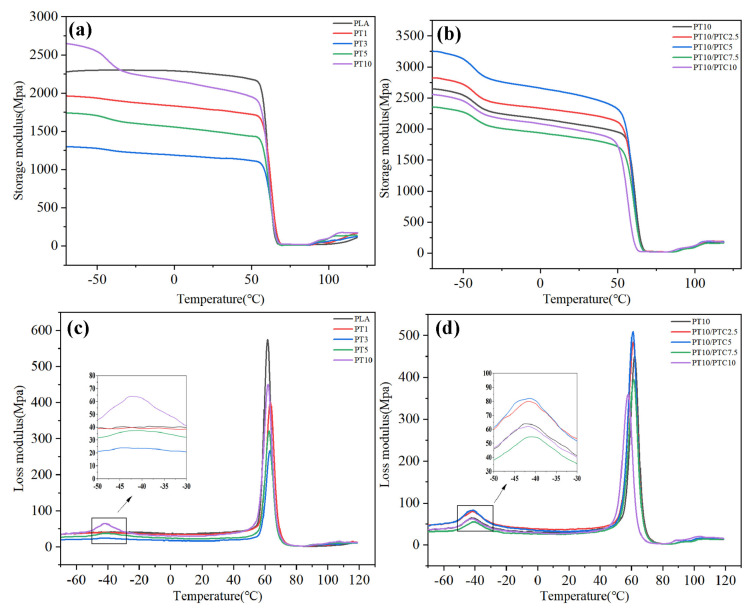
Storage modulus of (**a**) PLA/TPU and (**b**) PLA/TPU/PTC blends and loss modulus of (**c**) PLA/TPU and (**d**) PLA/TPU/PTC blends.

Figure 6 shows the storage modulus and loss modulus of PLA/TPU and PLA/TPU/PTC blends as a function of temperature.

It can be seen from Figure 6a that the addition of TPU reduces the storage modulus of PLA due to the plasticizing effect of TPU leading to the reduction of stiffness of PLA. In Figure 6b, the storage modulus of PT10/PTC2.5 and PT10/PTC5 are higher than that of PT10, suggesting that PTC as compatibilizer could promote the dispersion of TPU phase and reduce its deterioration effect on the stiffness of the blend.

There are two peaks in the loss modulus curves of the blends, which represent the α relaxation of the molecular chain motion of PLA and TPU respectively, i.e., the glass transition temperature. As shown in Figure 6c,d, all the blends showed two *T*_g_s, corresponding to the *T*_g_ of TPU (about −40 °C) and PLA (about 65 °C). This indicated that PLA and TPU are immiscible. Notably, with the incorporation of PTC, the two *T*_g_s difference decreased from 110 °C to 105 °C, suggesting the compatibilization between PLA and TPU had been improved.

## 4. Conclusions

In this work, PTC was prepared by in situ ring-opening coordination polymerization, and then was used as a compatibilizer to improve the mechanical properties of PLA/TPU blends. FTIR and ^1^H-NMR confirmed that PTC prepared by in situ ring-opening polymerization was indeed a copolymer. The PLA/TPU blends without PTC showed a conspicuous two-phase structure and poor interface adhesion. When the amount of TPU was 10 wt.%, the impact strength of PLA/TPU blend was only 4.7 kJ/m^2^ higher than that of pure PLA, indicating a very limited improvement in mechanical properties. In order to further improve the interface adhesion and mechanical properties of PLA/10TPU blends, PLA/TPU/PTC blends were prepared with varying contents of PTC. The addition of PTC reduced the size of TPU particles and the TPU particles were more evenly distributed in the PLA matrix. When the PTC content was 5 wt.%, the PLA/TPU/PTC blend enhanced by 68.5% compared with pure PLA, and moreover, there was no significant change in the strength and stiffness. Moreover, the results of DMA were basically consistent with the mechanical properties and confirmed that PTC could improve the compatibility between PLA and TPU. Consequently, when PLA:TPU:PTC ratio was 90:10:5, the PLA/TPU/PTC blend exhibited better strength, rigidity, and toughness, suggesting the potential of the blends in the applications of biomedicine, auto parts, and tissue engineering.

## Figures and Tables

**Table 1:** Formulation of PLA/TPU and PLA/TPU/PTC blends.

Sample	PLA/wt.%	TPU/wt.%	PTC/wt.%
PLA	100	0	0
PT1	99	1	0
PT3	97	3	0
PT5	95	5	0
PT10	90	10	0
PT10/PTC2.5	90	10	2.5
PT10/PTC5	90	10	5
PT10/PTC7.5	90	10	7.5
PT10/PTC10	90	10	10

**Table 2:** Mechanical properties of PLA/TPU blends and PLA/TPU/PTC blends.

Sample	Tensile Strength (MPa)	Young’s Modulus (MPa)	Impact Strength (kJ/m^2^)
PLA	65.2 ± 1.0	5567 ± 101	16.5 ± 2.1
PT1	66.3 ± 2.2	5459 ± 206	16.9 ± 3.3
PT3	63.2 ± 2.6	5121 ± 246	19.2 ± 1.2
PT5	61.5 ± 2.0	4963 ± 102	19.4 ± 1.1
PT10	58.6 ± 3.3	4748 ± 200	21.2 ± 1.6
PT10/PTC2.5	66.7 ± 1.7	5308 ± 303	20.9 ± 2.8
PT10/PTC5	66.1 ± 2.0	5180 ± 204	27.8 ± 2.0
PT10/PTC7.5	61.6 ± 2.5	5205 ± 100	23.4 ± 2.5
PT10/PTC10	61.4 ± 3.1	5306 ± 261	21.0 ± 3.0

## Data Availability

Not applicable.
